# Hypoglycemia and Medical Expenses in Patients with Type 2 Diabetes Mellitus: An Analysis Based on the Korea National Diabetes Program Cohort

**DOI:** 10.1371/journal.pone.0148630

**Published:** 2016-02-18

**Authors:** Sang Youl Rhee, Soo Min Hong, Suk Chon, Kyu Jeung Ahn, Sung Hoon Kim, Sei Hyun Baik, Yong Soo Park, Moon Suk Nam, Kwan Woo Lee, Jeong-Taek Woo, Young Seol Kim

**Affiliations:** 1 Department of Endocrinology and Metabolism, Kyung Hee University School of Medicine, Seoul, Korea; 2 Department of Endocrinology and Metabolism, Graduate School of Medicine, Kyung Hee University, Seoul, Korea; 3 Division of Endocrinology and Metabolism, Department of Internal Medicine, Cheil General Hospital & Women's Healthcare Center, Dankook University College of Medicine, Seoul, Korea; 4 Division of Endocrinology and Metabolism, Department of Internal Medicine, Korea University College of Medicine, Seoul, Korea; 5 Department of Internal Medicine, Hanyang University College of Medicine, Guri, Korea; 6 Department of Internal Medicine, Inha University School of Medicine, Incheon, Korea; 7 Department of Endocrinology and Metabolism, Ajou University School of Medicine, Suwon, Korea; Nanjing Medical University, CHINA

## Abstract

**Background and Aims:**

Hypoglycemia is one of the most important adverse events in individuals with type 2 diabetes mellitus (T2DM). However, hypoglycemia-related events are usually overlooked and have been documented less in clinical practice.

**Materials and Methods:**

We evaluated the incidence, clinical characteristics, and medical expenses of hypoglycemia related events in T2DM patients based on the Korea National Diabetes Program (KNDP), which is the largest multi-center, prospective cohort in Korea (n = 4,350). For accurate outcomes, the KNDP data were merged with claims data from the Health Insurance Review and Assessment Service (HIRA) of Korea.

**Results:**

During a median follow-up period of 3.23 years (95% CI: 3.14, 3.19), 88 subjects (2.02%) were newly diagnosed with hypoglycemia, and the incidence of hypoglycemia was 6.44 cases per 1,000 person-years (PY). Individuals with hypoglycemia were significantly older (59.7±10.7 vs. 53.3±10.4 years, *p* < 0.001), had more hospital visits (121.94±126.88 days/PY, *p* < 0.001), had a longer hospital stays (16.13±29.21 days/PY, *p* < 0.001), and incurred greater medical costs ($2,447.56±4,056.38 vs. $1,336.37±3,403.39 /PY, *p* < 0.001) than subjects without hypoglycemia.

**Conclusion:**

Hypoglycemia-related events were infrequently identified among the medical records of T2DM subjects. However, they were associated significantly with poor clinical outcomes, and thus, hypoglycemia could have a substantial burden on the Korean national healthcare system.

## Introduction

Approximately 4 million (12.4%) have diabetes mellitus among Korean adults aged ≥30 years in 2013; 14.5% of males and 10.4% of females [1]. And, 22.2% of adults had impaired fasting glucose [1]. Therefore, one-third of the Korean population is diabetic or pre-diabetic [1]. Approximately 6 million Korean individuals are estimated to be diabetic in 2050 [1]. The high incidence of diabetes is a global healthcare concern [2], and the number of diabetic complications increases concurrently with the prevalence of diabetes. This eventually leads to a decreased quality of life and increased medical expenditure [3,4]. Therefore, early screening and appropriate medical intervention strategies for diabetes are important for the prevention of overt diabetes, its complications, and its associated medical expenses.

Hypoglycemia is one of the most important adverse events in individuals with type 2 diabetes mellitus (T2DM). Hypoglycemia occurs when blood glucose levels are < 70 mg/dl (3.9 mmol/L) [5] and can lead to the development of handicaps and difficulties in daily life as well as impose a severe economic burden on the healthcare system [6,7]. However, hypoglycemia-related events had been overlooked and documented less frequently in clinical practice.

Thus, the aim of the current study is to investigate the prevalence of hypoglycemia and the associated healthcare costs in Korean subjects with T2DM using data from the Korea National Diabetes Program (KNDP) cohort. In previous studies, hypoglycemic events in diabetic patients were correlated with recurrent physical and psychological morbidity and mortality, such as cardiovascular disease and cognitive dysfunction [8–10]. However, real clinical evidence of hypoglycemia-associated events in Korea is very rare. Therefore, we investigated the correlation between hypoglycemia and medical expenditure in Korean individuals with T2DM.

## Materials and Methods

### Data source

The KNDP cohort database was used for the present study. The KNDP is a nationwide, large-scale, prospective, multi-center cohort study comprising data from university hospitals in Korea for Korean type 2 diabetic patients and those at a high-risk for developing diabetes.

A total of 4,350 subjects was recruited from 12 university hospitals (Kyung Hee University Hospital, Kyung Hee University Hospital at Gangdong, Korea University Guro Hospital, Ajou University Hospital, Inha University Hospital, Hanyang University Guri Hospital, Gachon University Gil Medical Center, Pusan National University Hospital, Catholic Kwandong University Cheil General Hospital and Women’s Healthcare Center, Yeungnam University Medical Center, Inje University Sanggye Paik Hospital, and Hallym University Gangdong Sacred Heart Hospital) [11]; all patients were managed by specialists according to standard practical guidelines.

### Study population

**Fig 1:**
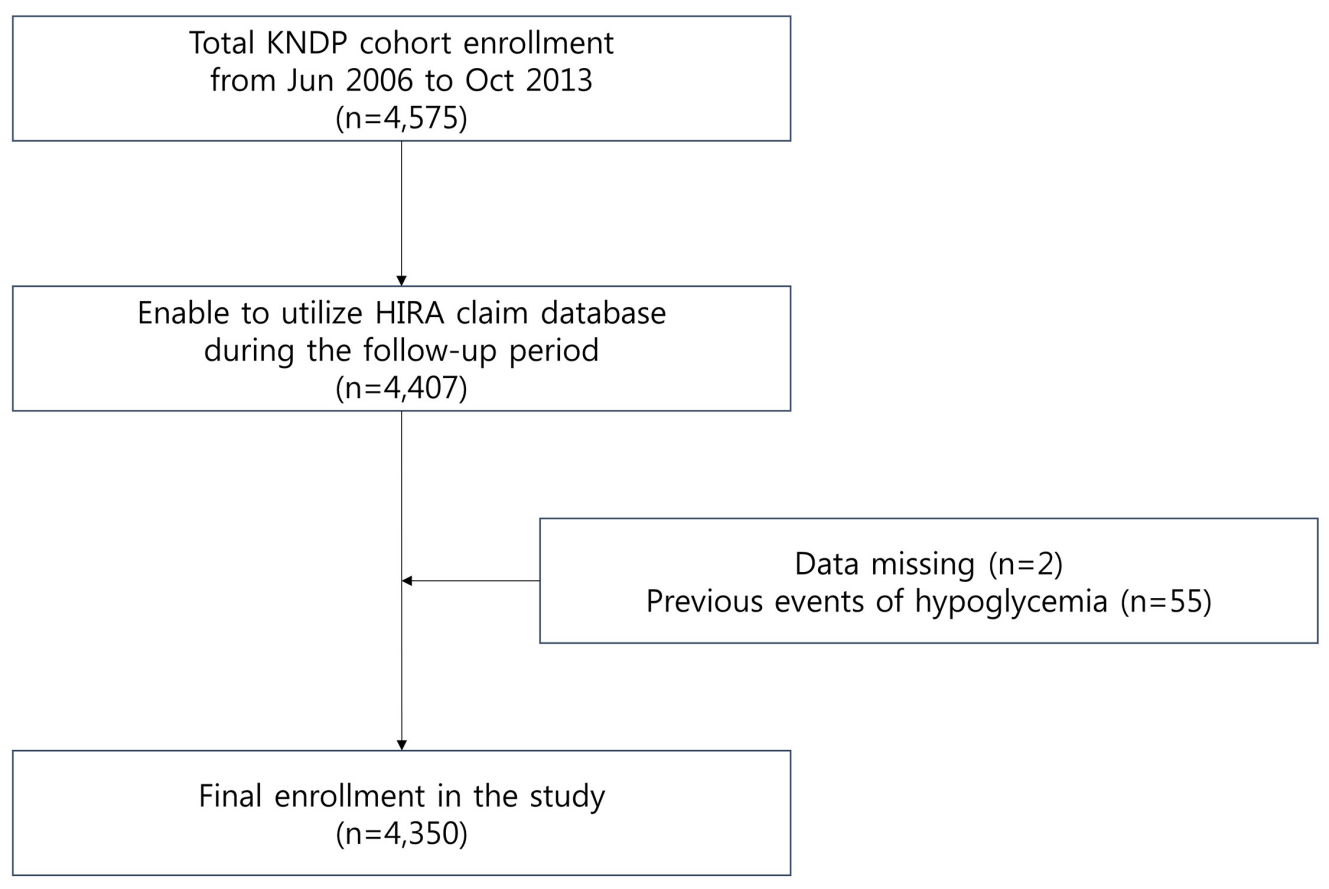
Subjects with T2DM enrolled based on the Korea National Diabetes Program (KNDP) cohort data.

As described in detail previously [11], the first patient was enrolled in Jun 2006, and primary observation of the registered participants ended in March 2014. The present study population consisted of 4575 individuals from the KNDP cohort whose data could be verified by merging with the Health Insurance Review and Assessment Service (HIRA) claim database. These data, which were collected between January 1, 2006 and December 31, 2010, were available for study because the HIRA claim database was open to external investigators for 5 years. When the HIRA database was merged with the KNDP data, 4407 patients for whom accurate personal information was available were selected in October 2013. Two patients were excluded because of missing values, and 55 patients were excluded because of previously diagnosed hypoglycemia. Finally, 4350 patients were included in the present study **(Fig 1)**.

### Study methods

The KNDP cohort data provided baseline clinical characteristics such as age, gender, blood pressure, body mass index (BMI), and habits of smoking and drinking, comorbidities such as hypertension, dyslipidemia, cardiovascular diseases, and cerebrovascular diseases, and histories of medication use, glucose levels, glycated hemoglobin (HbA1c) levels, liver function, and duration of T2DM. The baseline data mentioned above were collected from a survey performed at cohort registration and the patient’s medical records. The use of medication at baseline was defined as the initiation of a prescription within 3 months of enrollment and maintenance of the treatment for at least 2 months. The clinical and biochemical parameters were measured annually. The HIRA claim database provided the number of hospital visits, length of hospital stays, and the disease codes for hypoglycemia.

### Identification of hypoglycemia

Patients with hypoglycemia were identified based on a survey of the KNDP cohort and whether any of the claims codes from the HIRA database included ‘hypoglycemia’ as E10.63, E11.63, E12.63, E13.63, E14.63, E16.0, E16.1, or E16.2. These diagnostic codes of the 6^th^ version of the Korean Standard Classification of Diseases are compatible with the International Classification of Diseases-10.

### Statistical analyses

The incidence rates of hypoglycemia were calculated per 1000 person-years (PY). Descriptive statistics are expressed as means± standard deviation (SD.) and the clinical characteristics of subjects with and without hypoglycemia were compared using Student’s *t*-tests and chi-square tests. An analysis of covariance (ANCOVA) model was used to adjust for the effects of covariates, such as age, BMI, triglyceride levels, alanine aminotransferase (ALT) levels, comorbidity of cerebrovascular diseases, administration of oral hypoglycemic agents, duration of diabetes, follow-up period, recuperation period, and medical expenditures. All statistical analyses were performed using R software, version 3.0.3 and *p*-values < 0.05 were considered to be statistically significant.

### Ethics statement

All subjects submitted written informed consent and received sufficient explanation from relevant medical staff prior to participation in the study. The cohort data were registered at www.ClinicalTrials.gov (NCT00474838) and were approved by the institutional review boards of each hospital (IRB of Kyung Hee University Hospital, Kyung Hee University Hospital at Gangdong, Korea University Guro Hospital, Ajou University Hospital, Inha University Hospital, Hanyang University Guri Hospital, Gachon Medical School Gil Hospital, Pusan National University Hospital, Kwandong University Cheil General Hospital and Women’s Healthcare Center, Yeungnam University Medical Center, Inje University Sanggye Baik Hospital, and Hallym University Gangdong Sacred Heart Hospital).

## Results

### The incidence of hypoglycemia according to age

During the follow-up period, 88 subjects in the cohort experienced hypoglycemic events. The subjects with hypoglycemia were classified by age group, and the median follow-up period and incidence of hypoglycemia were documented. The median follow-up period for all enrolled subjects was 3.23 years (95% CI: 3.14, 3.19). The incidence of hypoglycemia was 3.72, 4.26, 9.34, and 25.75 cases per 1000 PY in patients aged <50, 50–59, 60–69, and >70 years, respectively **(Table 1)**.

**Table 1:** Incidence of hypoglycemia by age group.

Age (years)	<50	50–59	60–69	>70	Total
N	1,534	1,561	997	258	4,350
Median follow-up period	3.20	3.22	3.30	3.34	3.23
(years, 95% CI)	(3.13,3.21)	(3.14,3.22)	(3.10,3.20)	(3.02,3.25)	(3.14,3.19)
Incidence of hypoglycemia	18	21	29	20	88
(n, per 1000 person-years)	(3.72)	(4.26)	(9.34)	(25.75)	(6.44)

### The clinical characteristics of the study subjects with and without hypoglycemia

The study subjects were classified according to presence of hypoglycemic episodes, and the differences between the groups were assessed **(Table 2)**. At baseline, the age and mean disease duration were significantly higher in subjects who experienced hypoglycemia (*p*<0.001) compared with those who did not. There were no significant differences in BMI, waist circumference, systolic blood pressure, smoking or drinking habits, or comorbidities with hypertension, dyslipidemia, cardiovascular diseases, and cerebrovascular diseases between the two groups. Subjects who experienced hypoglycemia tended to use combination therapy for diabetes management more frequently as well as an increased proportion of renin-angiotensin system (RAS) blockade (*p* <0.001). Diastolic blood pressure, total cholesterol and low-density lipoprotein (LDL) cholesterol levels were significantly lower in hypoglycemic subjects. However, there were no significant differences in mean fasting glucose or HbA1c levels during the follow-up period between groups **(Table 2)**.

**Table 2:** Clinical characteristics of the study subjects.

	Hypoglycemia (-)	Hypoglycemia (+)	*p*	Total
N (n, %)	4,262 (98)	88 (2)		4,350 (100)
Age (years)	53.3±10.4	59.7±10.7	<0.001*	53.4±10.5
Men (n, %)	2,412 (56.6)	41 (46.6)	0.065	2,453 (56.4)
Duration of T2DM (years)	9.75±6.44	14.43±8.86	<0.001*	9.85±6.53
Smoking (n, %)				
current	335 (18.6)	14 (16.9)	0.270	356 (18.6)
ex	430 (23.9)	12 (14.5)		451 (23.5)
never	1,035 (57.5)	57 (68.7)		1,111 (57.9)
Medical history (n, %)				
hypertension	982 (53.0)	53 (63.1)	0.122	1,056 (53.6)
dyslipidemia	638 (34.5)	30 (35.7)	0.603	677 (34.4)
CVD + CVA	484 (26.1)	20 (23.8)	0.690	511 (25.9)
cardiovascular disease	284 (15.3)	13 (15.5)	0.577	300 (15.2)
cerebrovascular disease	257 (13.9)	9 (10.7)	0.673	270 (13.7)
Medications at baseline (n, %)				
metformin	625 (33.8)	36 (42.9)	0.109	676 (34.3)
sulfonylurea	799 (43.1)	39 (46.4)	0.445	856 (43.5)
insulin	202 (10.9)	9 (10.7)	0.779	216 (11.0)
other OHA	1,259 (68.0)	73 (86.9)	<0.001*	1,363 (69.2)
antiplatelet agents	941 (50.8)	45 (53.6)	0.861	1,004 (51.0)
RAS blockades	635 (34.3)	41 (48.8)	<0.001*	699 (35.5)
anti-hypertensive agents other than RAS blockades	912 (49.2)	47 (56.0)	0.371	978 (49.6)
statin	673 (36.3)	38 (45.2)	0.251	723 (36.7)
Anthropometry				
BMI (kg/m^2^)	25.1±3.1	25.0±2.9	0.796	25.1±3.1
waist circumference (cm)	88.6±7.9	88.4±6.8	0.642	88.6±7.8
SBP (mmHg)	127.7±15.7	128.0±15.5	0.082	127.8±15.8
DBP (mmHg)	77.6±9.9	74.8±8.7	0.037*	77.5±9.8
Laboratory findings				
fasting glucose (mg/dL)	138.5±45.0	132.0±41.7	0.310	138.4±45.2
HbA1c (%)	7.6±1.7	7.5±1.4	0.239	7.6±1.7
total cholesterol (mg/dL)	180.8±40.1	166.7±39.5	0.008*	180.2±40.0
triglyceride (mg/dL)	146.4±85.3	138.1±81.1	0.138	146.6±85.2
LDL cholesterol (mg/dL)	101.6±34.8	88.5±36.3	0.014*	101.0±35.0
HDL cholesterol (mg/dL)	47.7±12.6	48.2±12.6	0.837	47.7±12.6
BUN (mg/dL)	15.5±5.3	16.1±6.6	0.519	15.6±5.4
AST (IU/L)	25.6±17.7	27.9±15.0	0.158	25.6±17.5
ALT (IU/L)	26.5±19.1	29.6±19.8	0.167	26.6±19.1

Mean±S.D.

* *p*<0.05 as statistically significant by ANOVA or chi-square test. DM, diabetes mellitus; RAS, renin-angiotensin-aldosterone system; BMI, body mass index; SBP, systolic blood pressure; DBP, diastolic blood pressure; LDL, low- density lipoprotein; HDL, high density lipoprotein; BUN, blood urea nitrogen; AST, aspartate aminotransferase; ALT, alanine aminotransferase.

### Medical expenses and recuperation period (RECN) associated with hypoglycemia

The subjects who experienced hypoglycemia tended to incur more medical expenses ($2,447.56±4,056.38/PY) than the subjects who did not experience hypoglycemia ($1,336.37±3,403.39/PY) (*p*<0.05). (All costs presented in this paper are in US dollars (USD) using an exchange rate applicable at the end of the time of data collection, December 31, 2010, 1 USD = 1,120 Korean won)

**Fig 2:**
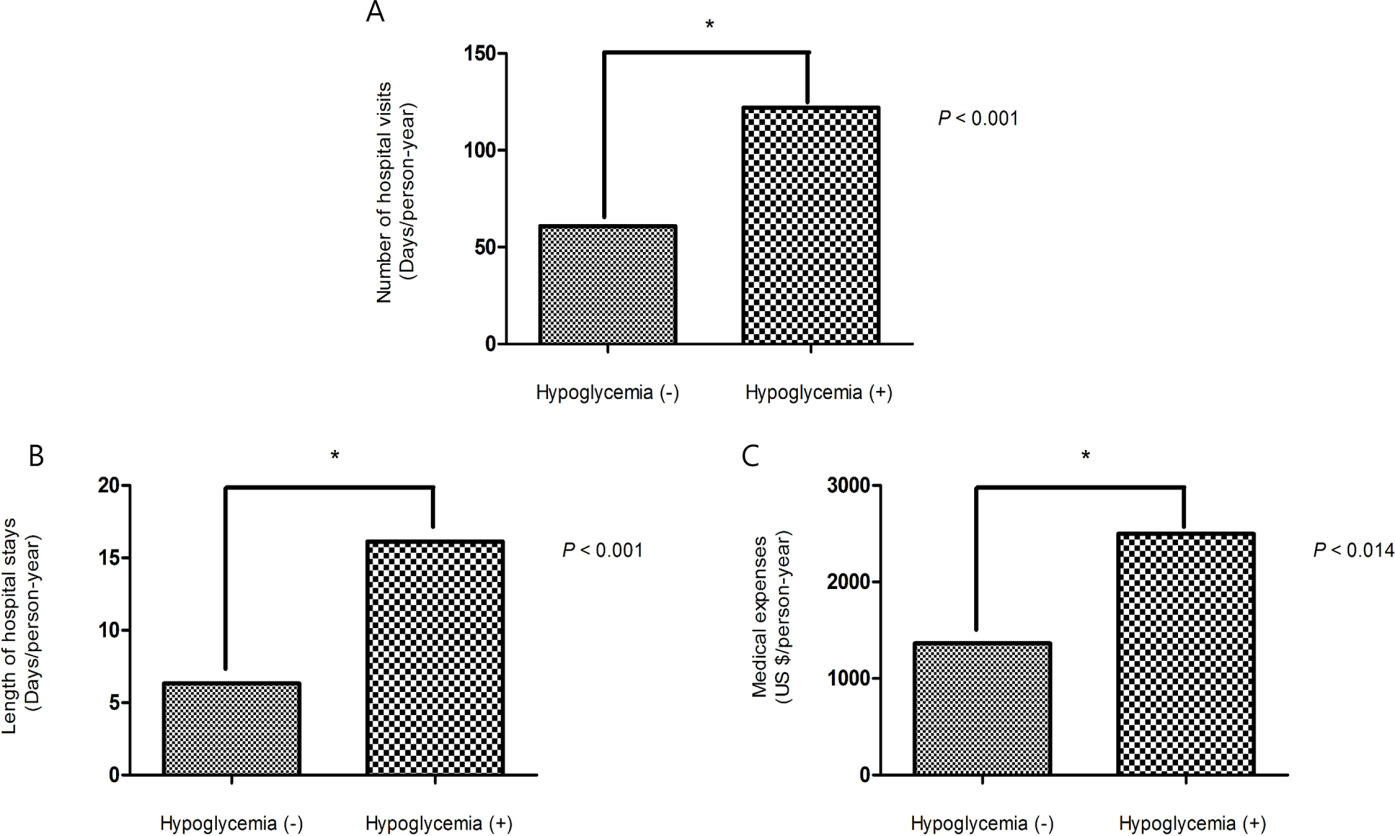
Comparisons between subjects with and without hypoglycemia after adjusting for age, follow-up period, duration of diabetes, recuperation period, BMI, cerebrovascular disease, DM medication, antihypertensive medication, TG, and ALT levels. (A) Number of hospital visits. (B) Length of hospital stays. (C) Medical expenses.

Additionally, subjects who experienced hypoglycemia tended to have more hospital visits (121.94±126.88 days/PY) and longer hospital stays (16.13±29.21 days/PY) than subjects who did not experience hypoglycemia **(Table 3)**. After adjusting for covariates such as age, follow-up period, T2DM duration, recuperation period, BMI, cerebrovascular disease, DM medications, antihypertensive medications, triglyceride (TG) levels, and ALT levels, hypoglycemia still contributed to a higher incidence of hospital visits, a longer duration of hospital stays and greater medical expenses (**Fig 2A, 2B and 2C).**

**Table 3:** Medical expenditures and recuperation periods of the study subjects.

	Hypoglycemia (-)	Hypoglycemia (+)	*p*	Total
	(n = 4262)	(n = 88)		(n = 4350)
Medical costs (US $ /PY)	$1,336.37±3,403.39	$2,447.56±4,056.38	0.012*	1,387±349
recuperation period (per PY)				
inpatients	6.33±21.44	16.13±29.21	0.002*	6.53±21.66
outpatients	60.76±95.95	121.94±126.88	<0.001*	62.00±97.03
total	67.09±101.06	138.06±132.75	<0.001*	68.53±102.27

Mean±S.D.

* *p*<0.05 as statistically significant by ANOVA. PY, person-years. All costs presented in this paper are in US dollars (USD) using an exchange rate applicable at the end of the time of data collection, December 31, 2010, 1 USD = 1,120 Korean won.

Moreover, medical expenditures and recuperation periods before and after hypoglycemic events in the hypoglycemia-experienced group were analyzed in the present study **(Table 4)**. Among subjects who experienced hypoglycemia, more medical expenses were incurred after hypoglycemic events ($3,365.14±8,318.19/PY) than before hypoglycemia events ($2,505.04±4,878.57/PY) (*p* = 0.18). And more hospital visits (147.62±250.77 days/PY) and longer hospital stays (21.25±39.73 days/PY) were incurred after hypoglycemia events than before hypoglycemia events (121.08±135.54 days/PY and 12.92±34.02 days/PY) (*p* = 0.25 and *p* = 0.04).

**Table 4:** Comparison of medical expenditures and recuperation periods before and after hypoglycemic events in the hypoglycemia experienced group.

	Before hypoglycemia	After hypoglycemia	*p*
Medical costs(US $ /PY)	$2,505.04±4,878.57	$3,365.14±8,318.19	0.18
recuperation period (per PY)			
inpatients	12.92±34.02	21.25±39.73	0.04*
outpatients	121.08±135.54	147.62±250.77	0.25
total	133.99±140.03	168.87±269.32	0.15

Mean±S.D.

* *p*<0.05 as statistically significant by ANOVA. PY, person-years. All costs presented in this paper are in US dollars (USD) using an exchange rate applicable at the end of the time of data collection, December 31, 2010, 1 USD = 1,120 Korean won.

## Discussion

The present study investigated the association between hypoglycemia and medical expenses using the KNDP cohort which is a nationwide, large-scale, prospective, and multi-center cohort study. To ensure accurate outcomes, nationally representative claims data from the HIRA Service of Korea was merged with the KNDP cohort. This facilitated a more precise assessment and more detailed outcomes of the cohort.

In summary, Korean subjects who experienced hypoglycemia were older, had longer disease duration, and had an increased tendency for the use of medication. However, the mean HbA1c levels during the follow-up period did not significantly differ between the groups with and without hypoglycemia. After multiple adjustments using an ANCOVA model, hypoglycemia remained significantly associated with hospital visits and the duration of stays as well as medical costs. These adjustments also demonstrated that the experience of hypoglycemic episodes was significantly associated with increased medical expenses in Korean subjects.

The incidence of hypoglycemia was 6.44 per 1000 PY among the 4,350 T2DM patients during a median follow-up period of 3.23 years. Hypoglycemic events tended to occur more frequently in older individuals and in those with a longer duration of T2DM. Hospital visits and longer stays were also more frequent in patients with hypoglycemia. Thus, hypoglycemia can potentially lead to excessive medical expenditure.

A prior survey (n = 1848) conducted in Germany, France, and the United Kingdom reported that 600 of the diabetic patients were worried about developing hypoglycemia [12]. In that study, the average frequencies of annual visits to the emergency room and of hospitalization were 0.65 and 0.47 per patient, respectively. Moreover, 10% of the patients stated that they had missed work in the past year due to hypoglycemia. Thus, the survey demonstrated that the diabetic patients had fears about hypoglycemia, and that hypoglycemia was found to be an emotional and economic burden on the medical system [12].

The expenditure related to hypoglycemic events in T2DM patients in Sweden was reported by Jonsson et al [13]. Of the 300,000 diabetic patients assessed in Sweden, approximately 27,000 reported experiencing hypoglycemic symptoms annually (0.09/PY). The total hypoglycemia-related medical expenditure was *€*4.25 million (€14/PY). Therefore, hypoglycemia accounted for a significant portion of the total medical costs in that study [13].

Some studies investigated the medical costs associated with hypoglycemic events based on the proportion of insurance claims which were stratified according to the type of resource (inpatients, outpatients, physician office, emergency department, and others) [14,15]. Inpatients accounted for 20% of claim data and 50% of the overall medical costs, which was the highest proportion of the total costs [14,15]. Similarly, Fabunmi et al. found that inpatients accounted for 87% of the overall medical costs [16]. Among these studies, the annual hypoglycemia-related medical costs in those treated with insulin were $1,528, compared to $620 for those treated with insulin analogues [14,15].

A study conducted in Germany reported that 95% of T2DM patients receiving emergency care for hypoglycemia, required hospitalization, and that the average length of hospitalization was 9.5 days [7].

Hypoglycemia contributes to direct and indirect medical costs. For example, hypoglycemia decreases work efficiency and productivity and, thus, indirectly generates non-medical costs, because expense loss occurs when working hours are reduced [13,17].

A Swedish study reported that the hiring rate of patients with hypoglycemia was relatively low [17]. Compared to diabetic patients without hypoglycemia who have a 30% employment rate, those with hypoglycemic symptoms had a lower employment rate at approximately 20%. When hypoglycemia-induced decreases in working hours and productivity were converted to indirect costs, the amount was $14.10 per month per person [17]. It has also been reported that direct and indirect hypoglycemia-related medical costs in Sweden were *€*4.25 million annually [12].

Several studies have assessed the burden of hypoglycemia in the United States and Europe [12,18]. Some of them demonstrated the hypoglycemia-related medical costs by resource type; inpatients, outpatients, emergency department, and reported the average length of hospitalization.

There are also meta-analysis articles for economic impact of hypoglycemia which performed literature reviews searching MEDLINE database using PubMed [19,20]. They reviewed direct and indirect costs of hypoglycemia in type 1 and 2 diabetic patients and in subgroups classified by the severity of hypoglycemia (with non-severe hypoglycemia events (NSHE), severe hypoglycemia events 1 (SHE1) who require non-medical, third party assistance, and severe hypoglycemia events 2 (SHE2) who require medical assistance) [19,20]. For example, direct costs of SHE2, SHE1, and NSHE were $1161, $66, and $11, respectively. Indirect costs related to hypoglycemia events were $242 in type 1 diabetic patients and $579 in type 2 diabetic patients [19].

However, this is the first study to examine hypoglycemia-related medical costs in adult Korean patients with T2DM, involving a large population of 4,350 subjects. The present study calculated the mean cost of hypoglycemic events, the number of hospital visits, and the length of hospital stays. Notably, not only the length of hospitalization but also the recuperation period that refers to the total period of treatment including the duration of drug ingestion or a course of injections was documented. Although the subject (hypoglycemia-related medical costs) has been studied before, there have been relatively few studies in Asia. The evidences generated from this study can be of important value to other future studies on the topic. Moreover, this is the first study to compare medical expenditures and recuperation periods before and after hypoglycemic events in the hypoglycemia-experienced group. There was no significant difference in medical expenditures and recuperation periods before and after hypoglycemic events in the hypoglycemia-experienced group. Nevertheless, the length of hospital stays after hypoglycemic events increased substantially (*p* = 0.04) in comparison to that before hypoglycemic events. This finding is meaningful because it decreases the QOL of diabetic patients and may eventually affect direct or indirect medical costs of diabetic patients who experienced hypoglycemic events. This point has not been investigated in previous studies and was supplemented in the paper as one of the novelties of this study.

Hypoglycemia-related events are infrequently observed among the medical records of T2DM subjects. However, hypoglycemia was significantly associated with poor clinical outcomes and, thus, could raise a substantial burden on the national healthcare system of Korea.

The assessment of hypoglycemia using the diagnostic codes from the HIRA database did not include all patients with hypoglycemia. And it is possible that the present study has a limitation of underestimating hypoglycemic events. When less severe hypoglycemic events that were not registered by the HIRA diagnostic codes, are considered, the hypoglycemia burden could be much greater.

Our investigation might be useful for the assessment of the economics of health care costs. And proper diabetic interventions could reduce hypoglycemic events and essentially the economic burden on the national healthcare system of Korea.
